# The Biological Activity and Composition of the Essential Oil of Sclerorhachis leptoclada (Asteraceae-Anthemideae) from Iran 

**Published:** 2014

**Authors:** Ali Sonboli, Mohammad Hossein Mirjalili, Javad Hadian, Morteza Yousefzadi

**Affiliations:** a *Department of Biology, Medicinal Plants and Drugs Research Institute, Shahid Beheshti University, G.C., Evin, 1983963113, Tehran, Iran. *; b *Department of Agriculture, Medicinal Plants and Drugs Research Institute, Shahid Beheshti University, G.C., Evin, 1983963113, Tehran, Iran. *; c *Department of Marine Biology, Hormozgan University, Bandar Abbas, Iran. *

**Keywords:** *Sclerorhachis*, Volatile oil, Antibacterial, Anticancer

## Abstract

The biological activity and composition of the essential oil of *Sclerorhachis leptoclada *Rech. f. an endemic species from northeast of Iran was studied. The essential oil was isolated from the aerial flowering parts of the plant and analyzed by GC and GC-MS. Fifty-four compounds accounting for 95.9% of the total oil were characterized. The main constituents were (*E*)-nerolidol (14.5%), terpinen-4-ol (13.3%), camphor (6.1%), 1,8-cineole (4.8%) and *p*-cymene (4.5%). The antimicrobial activity of the essential oil of *S. leptoclada *was tested against eight microbial strains and a fungi. The results of the bioassays showed that the Gram-positive bacteria*, Bacillus subtilis *and *Staphylococcus epidermidis*, were the most sensitive to the oil than others with the MIC value of 1.8 mg/mL. The tested fungi, *Saccharomyces cerevisiae *was highly inhibited by the oil of *S. leptoclada *with MIC value of 10 mg/mL. In the case of cytotoxicity, IC_50 _values estimated to be 312, 1250, 625 and 1250 μg oil/mL respectively, for the Vero, SW480, MCF7, and JET 3 cancer cell lines.

## Introduction

The genus *Sclerorhachis *(Rech. f.) Rech. f. belongs to the tribe Anthemideae of Asteraceae and consists of four species which are restricted to Iran and Afghanistan (1). In Flora of Iran, this genus is represented by two species distributed mainly in northeastern (2,3). *S. leptoclada *Rech. f. was first described by Rechinger (1981) from southern Khorasan as an endemic species from Iran. This species is characterized by its tender habit, rosette leaves with few short segments, extremely reduced stem leaves, and very small heads. *Sclerorhachis *species is confined to the most arid parts of the Iranian highlands (4). The composition and biological activity of several essential oils and extracts from different plant families have been studied (5,6). 

*Sclerorhachis leptoclada *with the common local name of «*Mastar*»**, **is an aromatic and very scented plant which aerial parts are used as a traditional herbal tea for the treatment of gastrointestinal disorders**, **also as flavouring agent for many kind of food products. The essential oil composition of another Iranian *Sclerorhachis *species *i.e*., *S. platyrachis *(Boiss.) Podlech. ex Rech. f. has already been studied (7). The main components in the oil of *S. platyrachis *were found to be α-pinene (31.2%), camphor (24.8%) and β-pinene (14.7%). Recently, inhibitory effect of the essential oils of *Sclerorhachis platyrachis *and *S. leptoclada *on phytopathogenic fungi, *Aspergillus flavus *and *Fusarium verticilloides *has been reported (8). As part of our study on the characterization of Iranian aromatic and medicinal plants here**, **composition, cytotoxicity and antimicrobial activity of the essential oil of *S. leptoclada *are investigated.

## Experimental

*Plant material*

The aerial parts of *S. leptoclada *were collected at full flowering stage in June 2009, from the Bejestan, Sarideh village at an altitude of 1250 m, Southern Khorasan province of Iran. A voucher specimen (MPH-1455) was deposited in the Medicinal Plants and Drugs Research Institute herbarium (MPH) of the Shahid Beheshti University of Tehran, Iran.

*Essential oil isolation procedure*

Air-dried aerial parts (100g) of *S. leptoclada *were subjected to hydro distillation for 3.5 h, using a Clevenger-type apparatus. The distilled was dried over anhydrous sodium sulfate and stored in a sealed vial at 4 ºC before analysis and test.

*Analytical procedure*

GC analysis was carried out on a Thermoquest-Finnigan Trace GC instrument equipped with a capillary DB-5 fused silica column (60 m × 0.25 mm *i.d*., film thickness 0.25 μm). Nitrogen (99.99% purity) was used as the carrier gas at the constant flow of 1.1 mL/min. The oven temperature was held at 60 ºC for 1 min, then programmed to 250 ºC at a rate of 4 ºC/min and held for 10 min. The injector and detector (FID) temperatures were kept at 250 ºC and 280 ºC, respectively. GC-MS analysis was performed on a Thermoquest-Finnigan Trace GC-MS apparatus, equipped with a DB-5 fused silica column (60m × 0.25 mm i.d., film thickness 0.25 μm). The oven temperature was raised from 60 ºC to 250 ºC at a rate of 5 ºC/min and held at 250 ºC for 10 min. The transfer line temperature was 250 ºC. Helium (99.99% purity) was used as the carrier gas at a flow rate of 1.1 mL/min; split ratio was 1/50. The quadrupole mass spectrometer was scanned over the 45-465 amu with an ionizing voltage of 70 eV and an ionization current of 150 μA. The constituents of the oil were identified by calculation of their retention indices under temperature-programmed conditions for *n*-alkanes (C_6_–C_24_; Merck, Germany) and the essential oil on DB-5 column. Identification of individual compounds was made by comparison of their mass spectra with those of the internal reference mass spectra from the library or with authentic compounds and confirmed by comparison of its retention indices with those reported in the literature (9).

*Antimicrobial assay*

For *antimicrobial assay *eight microbial strains were used: *Bacillus subtilis *(ATCC 465), *Staphylococcus aureus *(ATCC 25923), *S. epidermidis *(ATCC 12228), *Enterococcus faecalis *(ATCC 29737), *Escherichia coli *(ATCC 25922), *Klebsiella pneumoniae *(ATCC 10031), *Pseudomonas aeruginosa *(ATCC 85327), *Candida albicans *(ATCC 10231) and a fungi *Saccharomyces cerevisiae *(ATCC 9763). The antimicrobial activity of *S. leptoclada *essential oil was determined by the disk diffusion method (10). Briefly, 0.1 mL of a suspension of the test microorganism (108 cells/mL) was spread on Mueller-Hinton Agar plates for bacteria and Sabouraud Dextrose Agar**, **for the fungi. Sterile 6 mm disks, each containing 10 μL of essential oil were placed on the microbial lawns. Disks containing 10 μL of 1,8-cineole were used to study the antimicrobial activity of the oil main component. The plates were incubated at 37 ºC during 24 h for bacteria and at 30 ºC during 48 h for fungi. The diameters of the zones of inhibition were measured and reported in mm. Standard reference antibiotics Ampicillin and Gentamicin were used in order to control the sensitivity of the tested bacteria and Nystatine for fungi. All tests with three replications at weekly intervals were run in triplicate and mean values recorded. MIC values were determined by the broth micro dilution assay**, **recommended by the CLSI (11,12). Serial twofold dilutions of the essential oil were made in Mueller-Hinton Broth containing 0.5% Tween 80**, **for bacteria and Sabouraud Dextrose Broth with 0.5% Tween 80 for fungi**, **in 96-well micro titer plates. Fresh microbial suspensions prepared from overnight grown cultures**, **in the same media were added to give a final concentration of 5 × 105 organisms/mL. Controls of medium with either microbes or the essential oil alone were included. The micro plates were incubated at 37 °C during 24 h for bacteria and 30 °C during 48 h for fungi. The first dilution with no microbial growth was recorded as the MIC.

*Cell line and culture*

The Human colon adenocarcinoma (SW480), human breast adenocarcinoma (MCF7), choriocarcinoma (JET 3) and Monkey kidney (Vero) cell lines were obtained from Pasteur Institute of Iran. Cells were cultured in RPMI-1640 supplemented with 10% fetal bovine serum (Gibco) and 1% penicillin-streptomycin, at 37 ºC, in humidified air containing 5% CO2.

*Cytotoxicity assay*

Cytotoxicity was assessed by the tetrazolium-based colorimetric assay (MTT), which measures the reduction of the tetrazolium salt MTT (3-[4,5-dimethylthiazol-2-yl]-2, 5-diphenyltetrazolium bromide; Roche) into a blue formazan product, mainly by the activity of the mitochondrial enzymes, cytochrome oxidase and succinate dehydrogenase. Typically, 100 μL of cells suspension were plated at a density of approximate 2 ×104 cells per well in a 96-well plate, and were subsequently incubated at 37 ºC in a 5% CO2 humid incubator for 24 h. Then essential oils with different concentrations were added to each group (triplicate wells) and the incubation was continued for 24 h, followed by adding l0 μL (5 mg/mL) of MTT dye solution to each well for 4 h at 37 ºC. After removal of the MTT dye solution, cells were treated with 100 μL DMSO and the absorbance at 490 nm was quantified using ELISA reader. The cytotoxicity was calculated after comparing with the control (treated with 0.1% DMSO). Cytotoxicity is expressed as the concentration of drug inhibiting cell growth by 50% (IC_50_). All tests and analysis were run in triplicate and mean values recorded.

## Results and Discussion

*Essential oil analysis*

Hydro distilled essential oil of *S. leptoclada *gave a yellow oil with the yield of 1.1% (w/w) based on the dry weight of plant. Fifty-four compounds were identified, representing 95.9% of the total oil. The qualitative and quantitative compositions are presented in Table 1, where compounds are listed in order of their elution on the DB-5 column. The oil was characterized by a high concentration of oxygenated monoterpenes (39.0%) from which terpinen-4-ol (13.3%) being the major compound followed by camphor (6.1%) and 1,8-cineole (4.8%). Oxygenated sesquiterpenes constituted 26.8% of the total oil with (*E*)-nerolidol (14.5%) as the principal component. Monoterpene hydrocarbons comprised 16.1% of the oil in which *p*-cymene (4.5%) and γ-terpinene (3.9%) were found to be the major components of this fraction. Sesquiterpene hydrocarbons and other compound groups represent the 7.9% and 6.1% of the total oil, respectively. The literature survey on the analysis of the essential oils of *Sclerorhachis *species from Iran revealed that, only three main components of the oil of *S. leptoclada i.e*., bornyl acetate (18.8%), camphor (17.7%) and δ-cadinene (6.9%) have already been identified (8). Therefore, the present investigation reports the complete essential oil composition of *S. leptoclada *from endemic species of Iran´s flora for the first time. Regarding our results and previous report (8), it could be concluded that there is notable variation in the main constituents of the essential oils within *S. leptoclada *populations. The differences in chemical compositions could be attributed to several factors, including climatic conditions, geographical locations, time of collection, and developmental stage.

**Table 1:** Essential oil composition of *Sclerorhachis leptoclada*

**No.**	**Compound**	**RI** a	**LRI** b	**%**
1	Isobutyl isobutyrate	908	908	0.2
2	α-Thujene	929	924	0.5
3	α-Pinene	938	932	1.3
4	Camphene	955	946	0.8
5	Isoamyl propionate	965	960	0.7
6	Sabinene	977	969	1.3
7	β-Pinene	984	974	0.6
8	Butyl butanoate	999	993	0.5
9	Isoamyl isobutyrate	1008	1007	0.8
10	α-Terpinene	1021	1014	2.1
11	p-Cymene	1029	1022	4.5
12	Limonene	1033	1024	0.8
13	1,8-Cineole	1037	1026	4.8
14	(*Z*)-β-Ocimene	1046	1032	0.3
15	Prenyl isobutyrate	1049	1048	0.1
16	Isopentyl butanoate	1054	1052	0.5
17	γ-Terpinene	1063	1054	3.9
18	*cis*-Sabinene hydrate	1071	1065	1.2
19	Terpinolene	1093	1086	1.1
20	Linalool	1100	1095	1.5
21	2-Methyl butyl-2-methyl butyrate	1102	1100	3.2
22	*cis*-p-Menth-2-en-ol	1128	1118	0.8
23	Chrysanthenone	1131	1124	0.8
24	*trans*-p-Menth-2-en-ol	1146	1136	0.7
25	Camphor	1155	1141	6.1
26	Borneol	1177	1165	3.6
27	Terpinen-4-ol	1189	1174	13.3
28	α-Terpineol	1198	1186	2.2
29	Thymol, methyl ether	1236	1232	0.2
30	*cis*-Chrysanthenyl acetate	1265	1261	0.2
31	Lavandulyl acetate	1286	1288	0.3
32	Thymol	1290	1289	1.1
33	Terpinen-4-ol acetate	1304	1299	0.8
34	Neryl acetate	1361	1359	0.3
35	α-Copaene	1390	1374	1.2
36	(*Z*)-Jasmone	1397	1392	1.1
37	(*E*)-Caryophyllene	1434	1417	0.5
38	(*E*)-β-Farnesene	1455	1454	0.5
39	7-1,2-dehydro-Sesquicineole	1474	1471	0.3
40	*ar*-Curcumene	1488	1479	0.4
41	Germacrene D	1498	1484	1.3
42	β-Selinene	1504	1489	0.4
43	Bicyclogermacrene	1513	1500	0.8
44	Sesquicineole	1523	1515	1.4
45	δ-Cadinene	1535	1522	1.7
46	(E)-Nerolidol	1569	1561	14.5
47	Longipinanol	1579	1567	0.4
48	Spathulenol	1598	1577	2.2
49	Caryophyllene oxide	1604	1582	0.4
50	*allo*-Aromadendrene epoxide	1627	1639	0.6
51	α -Muurolol	1658	1640	1.7
52	α-Cadinol	1671	1652	3.2
53	α-Bisabolol	1693	1685	1.4
54	Amorpha-4,9-dien-2-ol	1706	1700	0.7
	Monoterpene hydrocarbons			16.1
	Oxygenated monoterpenes			39.0
	Sesquiterpene hydrocarbons			7.9
	Oxygenated sesquiterpenes			26.8
	Others			6.1
	Total			95.9

a RI, retention indices relative to C6–C24 *n*-alkanes on the DB-5 column;

b LRI, retention indices published in literature.

*Antimicrobial activity*

Table 2 shows *in-vitro *antimicrobial property of the essential oil of *S. leptoclada*. The oil showed potent to moderate inhibitory activity against tested microorganisms. The most sensitive microorganisms to the plant oil were found to be *Bacillus subtilis*, and *Staphylococcus epidermidis *with MIC value of 1.8 mg/mL. While *Pseudomonas aeruginosa *was the resistant Gram-negative strain*, Enterococcus faecalis *and *Klebsiella pneumonia *were found to be weakly inhibited by the oil tested. *Saccharomyces cerevisiae *was highly inhibited by the oil of *S. leptoclada *with MIC value of 10 mg/mL. Antifungal property of the essential oil obtained from *S. leptoclada *has already been investigated (8). The results revealed that the percentages of hyphal radial growth inhibition on *F. verticilloides *and *A. flavus *were 72.2 and 71.1 % by the essential oil of *S. leptoclada*. In addition, the maximum and minimum reductions of dry mycelium weights were 78.6% and 30.5%, respectively from the essential oil of *S. leptoclada *on the *A. flavus *and *F.verticilloide *fungi*.*

**Table 2:** Antimicrobial activity of the essential oil from *Sclerorhachis leptoclada*

**Microorganism**	**MIC** a	**IZ** b			
	**Essential oil**	**Essential oil** **(10 μL/disk)**	**Gentamicin** **(10 μg/disk)**	**Ampicillin** **(10 μg/disk)**	**Nystatine** **(30 μg/disk)**
*Bacillus subtilis*	1.8	25 ± 0.3	nt	14 ± 0.4	nt
*Enterococcus faecalis*	15	12 ± 0.4	nt	11 ± 0.3	nt
*Staphylococcus aureus*	3.75	18 ± 0.5	nt	13 ± 0.3	nt
*Staphylococcus epidermidis*	1.8	28 ± 0.3	nt	19 ± 0.5	nt
*Escherichia coli*	3.75	18 ± 0.2	23 ± 0.4	nt	nt
*Klebsiella pneumoniae*	>15	10 ± 0.3	20 ± 0.4	nt	nt
*Pseudomonas aeruginosa*	nt	-	12 ± 0.3	nt	nt
*Candida albicans*	>10	10 ± 0.3	nt	nt	18 ± 0.5
*Saccharomyces cerevisiae*	10	15 ± 0.6	nt	nt	18 ± 0.2

nt: not tested; moderately active (7-13); highly active (>14).

a Minimum inhibitory concentration presented as mg/mL.

b Inhibition zone includes diameter of disk (6 mm). Inactive (-);

*Cytotoxicity activity*

To evaluate the cytotoxic effect of the test essential oil against commonly used cancer cell lines (human and monkey), assays were performed using MTT reduction as the critical endpoint. Growth of all four cell lines*, i.e*., human colon adenocarcinoma (SW480), breast adenocarcinoma (MCF7), and choriocarcinoma (JET 3), as well as monkey kidney (Vero) cells, was inhibited in a dose-related manner after 24 h of exposure to the essential oil (Table 3). IC_50_ values estimated to be 312, 1250, 625 and 1250 μg oil/mL respectively, for Vero, SW480, MCF7, and JET 3 cells.

**Table 3:** Cytotoxicity activity of the essential oil from *Sclerorhachis leptoclada*

**Cell lines**	**IC**_50_ ** (μg/mL)** a
Choriocarcinoma cell line (JET 3)	1250 ± 6.9
Human colon adenocarcinoma cell line (SW480)	1250 ± 8.1
Human breast cancer cell lines (MCF 7)	625 ± 7.2
Monkey kidney (Vero)	312 ± 8.6

a IC_50_ values were expressed as the mean±S.D., determined from the results of MTT assay in triplicate experiments.

## Conclusions

In the essential oil of *S. leptoclada *oxygenated sesquiterpene, (*E*)-nerolidol (14.5%), oxygenated monoterpenes, terpinen-4-ol (13.3%) and camphor (6.1%) were found to be the principal constituents, while in the essential oil of *S. platyrachis *monoterpene hydrocarbons, α-pinene (31.2%) and β-pinene (14.7%), and oxygenated monoterpene camphor (24.8%) have already been characterized as major compounds (7). It is conceivable that the activity of the oil from *S. leptoclada *could be attributed mainly to the presence of (*E*)-nerolidol and terpinen-4-ol, which their biological activity has already been elaborated (13,14). The essential oil composition and the observed antimicrobial properties support the ethnobotanical uses of this plant in the folk medicine of southeastern Iran as an herbal tea. But, further phytochemical analysis, identification of active compounds and its applications in the field of health could be suggested for future investigations.
